# Stuck in Stage 3: The Case of an Effective Depression Intervention for African American Older Adults

**DOI:** 10.1093/geroni/igab046.1234

**Published:** 2021-12-17

**Authors:** Laura Gitlin

**Affiliations:** Drexel University, College of Nursing and Health Professions, Drexel University, Pennsylvania, United States

## Abstract

Beat the Blues (BTB) is a culturally tailored depression program for older African Americans. Tested in an NIA Stage 3 efficacy trial, findings showed statistically and clinically significant benefits, including decreased depressive symptoms, improved depression knowledge and symptom recognition, and behavioral activation. The multi-component intervention was co-constructed in partnership with a large senior center. Drawn from previously tested depression programs and tailored to preferences/needs of the targeted population, its five components included care management, depression education and symptom recognition, resources/referrals, and stress reduction and behavioral activation techniques. Despite significant findings, strong effect sizes and high acceptability, moving BTB to NIA Stage 4 (effectiveness) or 5 (dissemination) has been challenging. Challenges that will be discussed include lack of senior center funding to support training and delivery and infrastructure to embed BTB in community-based programs, and reluctance of health systems to adopt BTB because of its focus on one racial group.

